# Associations of adolescents’ lifestyle habits with their daytime functioning in Japan

**DOI:** 10.5935/1984-0063.20190151

**Published:** 2020

**Authors:** Jun Kohyama

**Affiliations:** Tokyo Bay Urayasu Ichikawa Medical Centre, Sleep medicine - Urayasu - Chiba - Japan

**Keywords:** Screen Time, Academic Performance, Sleepiness, Breakfast

## Abstract

**Objective:**

To assess associations of adolescents’ lifestyle habits with their daytime functioning in Japan.

**Methods:**

A total of 2,722 questionnaires obtained from pupils in grades 5 to 12 in Japan were assessed by the multiple comparison test to determine significant differences in the lifestyle habits among the self-reported academic performance categories (AP1: very good; AP2: good; AP3: not good; AP4: poor).

**Results:**

The average non-school-day screen time of AP4 pupils was significantly longer than that of AP1 pupils in elementary and junior high schools. In junior and senior high schools, AP4 pupils showed more sleepiness and higher occurrence of breakfast skipping than AP2 pupils. In all school types, sleep duration showed no significant differences among the self-reported academic performance categories.

**Discussion:**

Avoiding sleepiness, breakfast skipping, and heavy media usage is expected to ensure adolescents’ daytime functioning. Although not studied here, napping might improve adolescents’ daytime functioning.

## Introduction

Lifestyle habits affect variable aspects of health and daytime functioning^1-3^. For example, regular meal patterns, intake of healthy food items, and physical activity, in addition to sleep and body mass index (BMI), are known to affect academic performance (AP)^1^. In addition, skipping breakfast, eating quickly, excessive eating, long hours of TV watching and video-game playing, physical inactivity, and short sleep duration were associated with being overweight in adolescence^2^. Interestingly, an inverse linear association was observed between BMI and AP^3^. In my previous article^4^, better self-reported AP was found to be associated with female gender, lower grade, less sleepiness, lower BMI, less breakfast skipping, less constipation, early non- school-day wake-up time, and short non-school-day screen time. In a famous study titled ‘Sleep schedules and daytime functioning in adolescents’^5^, better self-reported AP was reported to be associated with earlier bedtime, longer school-day sleep duration, and earlier non-school-day wake time by means of a multiple comparison test of the Bonferroni post-hoc method (MCTBM). However, in my previous report, sleep duration was not examined. In the current study, associations of variable lifestyle habits with self-reported AP were assessed by a similar method using the classic study^5^ among adolescents. According to Wolfson and Carskadon^5^, the term *daytime functioning* includes mood, school performance, and behaviour, and they assessed associations of sleep schedule with depressive mood, sleepiness, and sleep/wake behaviour problems. In the current study, however, sleepiness, physical activity, after-school activity, and AP were included in the term *daytime functioning*. In addition, sleepiness rather than sleep duration has recently been paid attention to in association with daytime functioning^6-8^. Associations among sleep duration, AP, and sleepiness were also assessed.

## Materials and Methods

The questionnaire (Table 1) was original^4^, drawn up by taking queries from the Japan Society of School Health^9^ into consideration. A questionnaire was delivered to each student in grades 5 to 12 by his or her school teachers between October 2016 and November 2018. No participant was overlapped. A letter was also delivered assuring the students that their responses would be treated anonymously and confidentially and that it was voluntary to participate. Written consent (signed by a guardian) and completed questionnaires were collected by school teachers on a different day and were subsequently sent to the author. Of the 4,208 questionnaires collected from 28 public schools (15 elementary schools [ES], 8 junior high schools [JHS], and 5 high schools [HS]), 2,722 agreed to participate in the study and answered all the required questions. In these schools, school start time ranged from 8:35 to 8:45 in ES, 8:10 to 8:25 in JHS, and 8:35 to 8:40 in HS, and end time from 15:10 to 15:45 in ES, 15:45 to 16:05 in JHS, and 15:40 to 16:10 in HS. No school was multi- shift, such as options of attending school in either the morning or the afternoon. None of the schools provided a napping period.

**Table 1:** Questionnaire.


**(1) Please mark your grade**
Elementary school (grade 5, 6) Middle high school (grade 1, 2, 3) High school (grade 1, 2, 3)

**(2) Please mark your gender**
Gender (male, female)

**(3). Please describe your height and weight.**
Height ( cm) Weight ( kg)

**(4). Please mark your bed time before school days.**
1. < before 8 PM, 2. 8PM-9PM, 3. 9PM-10PM, 4. 10PM-11PM, 5. 11PM-12AM, 6. 12AM-1AM, 7. 1AM-2AM, 8. 2AM-3AM, 9. >after 3AM

**(5). Please mark your bed time before non-school days.**
1. < before 8 PM, 2. 8PM-9PM, 3. 9PM-10PM, 4. 10PM-11PM, 5. 11PM-12AM, 6. 12AM-1AM, 7. 1AM-2AM, 8. 2AM-3AM, 9. > after 3 AM

**(6). Please mark your wake time on school days.**
1. < before 5AM, 2. 5AM-6AM, 3. 6AM-7AM, 4. 7AM-8AM, 5. 8AM-9AM, 6. 9AM-10AM, 7. 10AM-11AM, 8. 11AM-12PM, 9. > after 12PM

**(7) Please mark your wake time on non-school days.**
1. < before 5AM, 2. 5AM-6AM, 3. 6AM-7AM, 4. 7AM-8AM, 5. 8AM-9AM, 6. 9AM-10AM, 7. 10AM-11AM, 8. 11AM-12PM, 9. > after 12PM

**(8) Please mark the frequency you feel sleepy during class.**
1. Never 2. Sometimes 3. Often or 4. Always

**(9) Please mark your the frequency of eating breakfast.**
1. Always 2. Often 3. Sometimes or 4. Never

**(10) Please mark your the frequency of defecation.**
1. Every day, 2. Every other day, 3. Once every two to three days, 4. Twice a week or less

**(11) Please mark the time you usually take dinner.**
1. Around 6 PM, 2. Around 7 PM, 3. Around 8 PM, 4. Around 9 PM, 5. Around 10 PM, 6. Around 11 PM, 7, Later than 11 PM, 8. Not determined

**(12) Do you participate in any kinds kind of after-school activity?**
1. Yes 2. No

**(13) If yes, please mark your the frequency of participating in an after-school activity**
1. Once a week, 2. Twice a week, 3. Three times a week, 4. Four times a week, 5. Five times a week, 6. Six times a week, 7. Every day.

**(14) Please mark the average duration of a single after-school activity.**
1. 1 h, 2. 2 h, 3. 3 h, 4. 4 h, 5. 5 h or more.

**(15) How many days a week do you take a habitual exercise except for school lessons?**
0. No Zero -day per week, 1. One day per week, 2. Two days per week, 3. Three days per week, 4. Four days per week, 5. Five days per week, 6. Six days per week or 7. Seven days per week

**(16) How long do you use various media devices (television, video, video game, digital versatile disc, computer, tablet, mobile (cell) phone, and smart phone) in a day? Please answer separately on school days and non-school days**
On a school day. 1. < 2 h, 2. 2-4 h, 3. 4-6 h, 4. 6-8 h, 5. 8 h or more.
On a non-school day. 1. < 2 h, 2. 2-4 h, 3. 4-6 h, 4. 6-8 h, 5. 8 h or more.

**(17) Please mark one of the following choices where your over-all overall academic performance mostly belongbelongs to.**
1. Very good, 2. Good, 3. not goodAvergaeAverage, 4. Poor. In order t

The selected numbers in the query on sleepiness, skipping breakfast, and defecation are termed as the sleepiness score, skipping breakfast score, and defecation score, respectively. For dinner regularity, the choice of 1 to 7 was categorised into regular dinner (dinner regularity score of 1) and the eighth choice into irregular dinner (dinner regularity score of 2). Hours of after-school activity per week, obtained by the product of the following two numbers of the two queries: one on the frequency and the other on the duration, and number of days engaged in physical activity per week were also assessed.

To calculate sleep duration, we needed a representative time for each category of bed and wake times. Representative times for each bedtime category (1. < 8PM; 2. 8PM-9PM; 3. 9PM-10PM; 4. 10PM-11PM; 5. 11PM-12AM; 6. 12AM-1AM; 7. 1AM-2AM; 8. 2AM-3AM; 9. > 3AM) were determined as follows: 7:30PM, 8:30PM, 9:30PM, 10:30PM, 11:30PM, 12:30AM, 1:30AM, 2:30AM, and 3:30AM. For the wake time category (1. < 5AM; 2. 5AM-6AM; 3. 6AM-7AM; 4. 7AM-8AM; 5. 8AM-9AM; 6. 9AM-10AM; 7.10AM-11AM; 8. 11AM-12PM; 9. > 12PM), representative times were as follows: 4:30AM, 5:30AM, 6:30AM, 7:30AM, 8:30AM, 9:30AM, 10:30AM, 11:30AM, and 12:30PM. The nighttime sleep duration on school nights was calculated as the difference between bedtime before school days and wake time on school days of these representative times. The nighttime sleep duration on non-school nights was calculated as the difference between bedtime before non-school days and wake time on non-school days of these representative times. Nighttime sleep duration was categorised into six groups (1. less than 6 hours; 2. 6-7 hours; 3. 7-8 hours; 4. 8-9 hours; 5. 9-10 hours; 6. 10 hours or more). To calculate screen time, representative times for each screen time category (1. less than 2 hours; 2. 2-4 hours; 3. 4-6 hours; 4. 6-8 hours; 5. 8 hours or more) were determined as follows: 1 hour, 3 hours, 5 hours, 7 hours, and 9 hours.

Since BMI was reported to be altered markedly among school-aged children and adolescents in Japan[9], an analysis of variance was conducted to determine the differences among BMIs of 16 categories divided by gender and grade (male and female categories of grades 5 to 12). On analysis, these gender and grade standardised BMIs were used. To determine significant differences of lifestyle habits between self-reported AP categories (AP1: very good; AP2: good; AP3: not good; AP4: poor), the MCTBM was conducted. The MCTBM was also used to assess significant differences of sleepiness among sleep duration categories. For analysis of group differences by the MCTBM, in addition to a p-value of less than 0.05, effect sizes where two groups differ by more than one-third of the sample standard deviation (SD) are considered significant. This criterion (Cohen’s d value = 1/3), slightly lower than the midpoint between small (d=0.20) and medium (d=0.50) effect sizes, is the same one used in the above-mentioned classic study^5^. These analyses were conducted using software called ‘BellCurve for Excel’.

This study was approved by the Committee for Medical Research Ethics of Tokyo Bay Urayasu Ichikawa Medical Centre (no. 199). Part of this study has been published elsewhere^4^.

## Results

Table 2 shows the means and SDs of lifestyle variables in each school type with a pair showing significant difference among three school type categories. From ES to HS via JHS with enough effect sizes, the average bedtimes were significantly delayed in both school nights and non-school nights. The average nighttime sleep durations were significantly shortened in both school nights and non-school nights from ES to JHS via JHS. The average self-reported AP of ES was significantly better than that of JHS and HS. The average screen time of HS was significantly longer than that of ES in both school days and non-school days. The average sleepiness scores became significantly higher from ES to HS via JHS. The average weekly hours of after-school activity of HS were significantly higher than those of ES and JHS. The average BMI values became significantly higher from ES to HS via JHS.

**Table 2:** Means and standard deviations of lifestyle variables in each school type with a pair showing significant difference among three school type categories.

Lifestyle variables	School types (number of pupils)			A pair showing a significant difference (Cohen's d value)
	ES (956)	JHS (1,049)	HS (717)	
School-day wake time: mean (time)±SD (hr)	6:29±0.45	6:28±0.70	6:19±0.78	No pair shows significant differences
School night bedtime: mean (time)±SD (hr)	21:54±0.79	23:02±1.12	23:47±1.00	ES-JHS** (1.17)
				ES-HS** (2.08)
				JHS-HS** (0.69)
School night sleep duration: mean (hr)±SD (hr)	8.57±0.78	7.43±1.08	6.53±1.02	ES-JHS** (1.22)
				ES-HS** (2.24)
				JHS-HS** (0.85)
Non-school-day wake time: mean (time)±SD (hr)	7:36±1.12	7:58±1.50	8:05±1.68	ES-HS** (0.35)
Non-school night bedtime: mean (time)±SD (hr)	22:15±0.90	23:20±1.31	0:04±1.17	ES-JHS** (0.98)
				ES-HS** (1.75)
				JHS-HS** (0.59)
Non-school night sleep duration: mean (hr)±SD (hr)	9.35±1.06	8.63±1.35	8.02±1.54	ES-JHS** (0.60)
				ES-HS** (1.00)
				JHS-HS** (0.42)
Self-reported academic performance score: mean±SD	2.16±0.72	2.60±0.82	2.60±0.82	ES-JHS** (0.58)
				ES-HS** (0.57)
School-day screen time: mean (hour)±SD (hr)	2:01±1.35	2:12±1.63	3:05±1.91	ES-HS** (0.64)
				JHS-HS** (0.49)
Non-school-day screen time: mean (hr)±SD (hr)	3:22±1.99	3:42±2.22	4:22±2.30	ES-HS** (0.46)
Sleepiness score: mean±SD	1.59±0.65	2.00±0.74	2.51±0.85	ES-JHS** (0.59)
				ES-HS** (1.23)
				JHS-HS** (0.65)
Dinner regularity score: mean±SD	1.38±0.49	1.40±0.49	1.32±0.47	No pair shows significant differences
Defecation score: mean±SD	1.59±0.86	1.71±0.92	1.57±0.87	No pair shows significant differences
After-school activity (hr/week): mean (hr)±SD (hr)	5.05±5.78	4.49±5.54	9.20±10.19	ES-HS** (0.50)
				JHS-HS** (0.57)
Skipping breakfast score: mean±SD	1.11±0.36	1.23±0.58	1.34±0.73	ES-HS** (0.41)
Number of days/weeks engaged in habitual exercise: mean (day)±SD (day)	2.57±2.46	3.79±2.99	2.95±3.05	ES-JHS** (0.45)
Body mass index: mean±SD	18.0±2.84	19.3±2.77	20.3±2.41	ES-JHS** (0.47)
				ES-HS** (0.88)
				JHS-HS** (0.39)

Note: ES: elementary school; JHS: junior high school; HS: high school; SD: standard deviation; * *p*<0.05;

** *p*<0.01; hr: hour.

Significant gender differences were obtained in non-school-day wake time of ES pupils (females were significantly delayed), defecation score (females were significantly higher), and number of weekdays engaged in habitual exercise (females were significantly lower).

**Figure 1:**
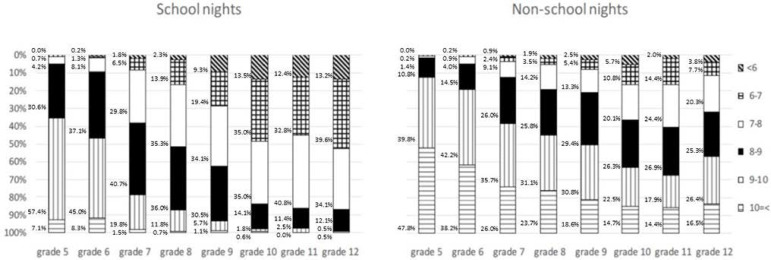
Distribution of sleep duration categories (less than 6 hours, 6-7 hours, 7-8 hours, 8-9 hours, 9-10 hours, 10 hours or more) in each grade (school nights [left] and nonschool nights [right]).

To see the details of the decreases of nighttime sleep durations from ES to HS, distributions of nighttime sleep duration categories (less than 6 hours, 6-7 hours, 7-8 hours, 8-9 hours, 9-10 hours, 10 hours or more) in each grade are displayed in Figure 1. Except for non-school nights of grade 12 pupils, the longest nighttime sleep duration category moved toward the shorter categories with grade progression.

To determine significant differences of lifestyle habits between self-reported AP categories, MCTBM was applied for all the 2,722 data obtained. Pupils who assessed themselves as belonging to the worst AP category (AP4) showed significantly later bedtimes for both school nights and non-school nights than those who believed themselves as belonging to the other three categories (AP1, AP2, and AP3). Similarly, AP4 pupils also significantly had the highest sleepiness and skipping breakfast scores than pupils of the other three categories (AP1, AP2, and AP3). The average nighttime sleep duration on school nights of AP4 pupils was significantly shorter than that of AP1 and AP2 pupils, while the mean screen times during both school days and non-school days of AP4 pupils were significantly longer than those of AP1 and AP2 pupils. In addition, the average non-school-day wake time of AP4 pupils was significantly later than that of AP1 pupils. Since these eight variables showing significant differences between self-reported AP categories also had school type differences (Table 2), a similar analysis using the MCTBM was conducted separately for each school type.

Table 3 shows the means and SDs of lifestyle variables in each self-reported AP category, with a pair showing a significant difference among four self-reported AP categories in each school type. In ES pupils, non-school-day screen time was significantly shorter in AP1 pupils than in AP3 and AP4 pupils. Among JHS pupils, AP4 pupils showed significantly later bedtimes on both school nights and non-school nights, longer screen times on both school nights and non-school nights, and higher skipping breakfast scores than pupils of the other three categories (AP1, AP2, and AP3). In addition, the average non-school-day wake time was significantly later in AP4 JHS pupils than in AP1 and AP2 JHS pupils, and the mean sleepiness score was significantly higher in AP4 JHS pupils than in AP2 JHS pupils. In HS, a significantly higher average sleepiness score was seen in AP4 pupils than in pupils of the other three categories, and the average skipping breakfast score was significantly higher in AP4 pupils than in AP2 pupils. It should be noted that no pair among the four self-reported AP categories in all three school types showed significant differences in nighttime sleep duration. Hours of after-school activity per week also showed no significant difference among self-reported AP categories in the above-mentioned four MCTBM analyses.

**Table 3:** Means and standard deviations of lifestyle variables in each self-reported academic performance category with a pair showing significant difference among four self-reported academic performance categories in each school type.

School types and lifestyle variables	Self-reported academic performance categories				A pair showing a significant difference (Cohen's d value)
	[AP1: very good; AP2: good; AP3: not good; AP4: poor] (number of pupils)				
ES	AP1 (153)	AP2 (525)	AP3 (246)	AP4 (32)	
Non-school-day screen time: mean (hr)±SD (hr)	2:59±2.03	3:16±1.91	3:44±2.02	4:00±2.38	AP1-AP3** (0.38)
					AP1-AP4* (0.46)
JHS	AP1 (79)	AP2 (403)	AP3 (422)	AP4 (145)	
School night bedtime: mean (time)±SD (hr)	22:58±1.21	22:54±1.02	23:02±1.06	23:28±1.41	AP1-AP4** (0.38)
					AP2-AP4** (0.46)
					AP3-AP4** (0.34)
Non-school-day wake time: mean (time)±SD (hr)	7:40±1.33	8:00±1.38	8:02±.50	8:23±.82	AP1-AP4** (0.45)
					AP2-AP4** (0.36)
Non-school night bedtime: mean (time)±SD (hr)	23:05±1.38	23:08±1.18	23:23±1.24	23:53±1.60	AP1-AP4** (0.54)
					AP2-AP4** (0.53)
					AP3-AP4** (0.35)
School-day screen time: mean (hr)±SD (hr)	1:47±1.55	2:00±1.36	2:12±1.55	3:01±2.25	AP1-AP4** (0.64)
					AP2-AP4** (0.56)
					AP3-AP4** (0.43)
Non-school-day screen time: mean (hr)±SD (hr)	3:28±2.33	3:28±2.02	3:42±2.15	4:35±2.70	AP1-AP4** (0.45)
					AP2-AP4** (0.47)
					AP3-AP4** (0.36)
Sleepiness score: mean±SD	1.97±0.82	1.88±0.69	2.04±0.70	2.25±0.87	AP2-AP4** (0.47)
Skipping breakfast score: mean±SD	1.09±0.40	1.11±0.38	1.25±0.56	1.57±0.93	AP1-AP4** (0.68)
					AP2-AP4** (0.66)
					AP3-AP4** (0.42)
HS	AP1 (56)	AP2 (274)	AP3 (287)	AP4 (100)	
Sleepiness score: mean±SD	2.34±0.88	2.36±0.77	2.56±0.81	2.92±0.97	AP1-AP4** (0.63)
					AP2-AP4** (0.64)
					AP3-AP4** (0.40)
Skipping breakfast score: mean±SD	1.32±0.74	1.24±0.60	1.36±0.72	1.59±0.98	AP2-AP4** (0.43)

Note: ES: elementary school; JHS: junior high school; HS: high school;

* *p*<0.05;

** *p*<0.01.

As for nighttime sleep duration on school nights, the sleepiness score was significantly higher in category 1 (mean value ± SD; 2.61±0.83) than in categories 3 (2.17±0.82), 4 (1.85±0.70), 5 (1.59±0.65), and 6 (1.63±0.70); in categories 2 (2.45±0.88) and 3 than in categories 4, 5, and 6; and in category 4 than in category 5, respectively. On non-school nights, the sleepiness score was significantly higher in categories 1 (2.63±0.95) and 2 (2.42±0.84) than in 4 (2.08±0.82), 5 (1.85±0.77), and 6 (1.85±0.76), and in category 3 (2.28±0.88) than in 5 and 6 for nighttime sleep duration.

## Discussion

The association between better AP and a long sleep duration is widely acknowledged^1,5^. Consistent with these reports, the current first analysis using all data together revealed that the average nighttime sleep duration on school nights of AP4 pupils was significantly shorter than that of AP1 and AP2 pupils. However, further analysis conducted separately for each school type found no significant differences of nighttime sleep duration among the four self-reported AP categories in all three school types, although a lack of investigation on daytime sleep duration might contribute to this unexpected result. However, it should be noted that the need for sleep has individual variabilities, which are influenced by genetic, behavioural, medical, and environmental factors^10^. Moreover, sleepiness was recently reported to be a stronger predictor of AP than sleep duration^6,7^. In addition, a shortage of sleep duration was reported to be associated with structural changes and greater functioning^11^; shorter sleep duration was associated not only with lower mean diffusivity, which is generally associated with more neural tissues in widespread areas of the brain, but also with greater persistence and executive functioning (lower Stroop interference). Sleepiness is also known to impact all areas of adolescent functioning, including AP in addition to insufficient sleep^8^. Consistently, the present study showed that sleepiness score revealed significant differences between self-reported AP categories in JHS (AP2<AP4) and HS (AP1/AP2/AP3<AP4). Interestingly, sleepiness, instead of sleep duration, has received attention in terms of adolescents’ self-regulation; impairment in self-regulation is one of the mechanisms that leads to the adverse effects of insufficient sleep on health and functioning, and sleepiness instead of sleep duration was found to be associated with lower self-regulation among adolescents^12^. In the present study, the sleepiness score showed significant differences among nighttime sleep duration categories, but it did not reveal a higher frequency of sleepiness with short sleep duration or long sleep duration compared to those with normal sleep duration, as Alves et al. reported^13^. Further variable issues on sleepiness remain to be clarified. Anyway, more attention should be paid to sleepiness, though a subjective measure, in regard to adolescents’ daytime functioning, including AP and self-regulation.

Both a delayed school start time^5^ and napping^14^ are assumed to be possible tools to help adolescents with sleepiness. According to Uchimura^14^, after the introduction of a 15-minute nap in a HS, the three-year-average point of the National Centre Test for University Admissions of the HS against the national average point was increased in comparison with that point before nap introduction. Uchimura also reported that the HS students reported the improvement of alertness after nap introduction^14^. Napping might be a tool to help adolescents with daytime functioning.

The current result regarding skipping breakfast score in JHS and HS pupils is consistent with previous reports, showing an association between regular breakfast consumption and better AP^15^. Also, non-school- day screen time of AP4 pupils was found to be significantly longer than that of AP1 pupils in ES and JHS. The average school-day screen time of JHS AP4 pupils was also significantly longer than that of the other three categories. Information on the influence of screen time on functioning has recently increased, and Takeuchi et al.^16^ concluded that frequent Internet use is associated with decreased verbal intelligence and the development of smaller grey matter volume at later stages. To ensure their health and daytime functioning, we should prevent adolescents from engaging in heavy media usage and encourage avoiding sleepiness and breakfast skipping. However, it remains to be studied why the current study did not demonstrate associations between AP and BMI^3^.

After-school activity showed no significant difference among AP categories in all school types. In Japan, to improve their AP, 41.3% of middle HS pupils and 27.2% of HS pupils engage in private cram schools^17^. However, the current results do not support the widely believed notion in Japan that after-school activity is important to improve AP.

The survey^9^ using the queries we referred to has been conducted every two years since 1993, with several revisions by experts, and the results have been used as the fundamental data for policy-making and compiling manuals on proper lifestyles of children in Japan. The queries^9^ are assumed to be generalised and established.

This study has several limitations. First, this study lacked age information. This is because the queries^9^ we referred to had no age information. However, it should not be forgotten that age is an important biological factor. Second, the current study is a cross-sectional one. Therefore, no causal link could be discussed. Third, this study depended on questionnaire answers and lacked objective data. However, self-reported AP is found to be an accurate indicator of actual AP, although its limitations should be recognised^18^. Finally, this study did not assess socioeconomic status. Low socioeconomic status might be closely associated with biological sleep problems during development, probably due to nutritional, hygienic, and educational problems. Indeed, children from a low socioeconomic status^19^ show higher rates of sleep problems, such as short sleep duration, although the opposite result has been reported^20^. An association between socioeconomic status and sleep habits remains to be studied.

In summary, no significant differences of nighttime sleep duration were found among the four self-reported AP categories in all three school types, while the sleepiness score revealed significant differences between self-reported AP categories in JHS and HS. The average skipping breakfast score was significantly higher in AP4 pupils than in the other three categories (JHS) or pupils in AP2 (HS). The average school-day screen time of JHS AP4 pupils was significantly longer than that of the other three categories, and non-school-day screen time of AP4 pupils was significantly longer than that of AP1 pupils in ES and JHS. To ensure adolescents’ daytime functioning, we should pay more attention to sleepiness, breakfast skipping, and heavy media usage rather than sleep duration. Napping might be a tool to help adolescents with daytime functioning.
